# Autologous stem cell transplantation in autoimmune arthritis restores immune homeostasis by renewal of the natural Treg compartment

**DOI:** 10.1186/1546-0096-9-S1-P183

**Published:** 2011-09-14

**Authors:** T van den Broek, E Delemarre, J Meerding, E Wehrens, F Broere, N Wulffraat, M Boes, B Prakken, F van Wijk

**Affiliations:** 1 Department of Pediatric Immunology, Wilhelmina Children's Hospital, University Medical Center Utrecht, The Netherlands; 2 Institute of Infectious Diseases and Immunology, Utrecht University, Utrecht, the Netherlands

## 

Autologous stem cell transplantation (ASCT) is a last resort treatment for refractory juvenile idiopathic arthritis (JIA). It has been shown to induce dramatic and long-term improvements, but the underlying mechanisms remain to be elucidated. We investigated the potential role of regulatory T cells (Treg) in the processes of immune reconstitution and re-establishment of immune tolerance following ASCT.

In a mouse model of proteoglycan-induced arthritis, (pseudo)ASCT following lethal irradiation reduced arthritis scores and restored the immune balance of pro-inflammatory effector T cells and Treg. Directly following the ASCT the majority of Treg consisted of Treg that survived the conditioning and had expanded vigorously. After a few weeks the infused stem cell-derived Treg started dominating the Treg pool and these “new” thymus-derived Treg showed more suppressive capacity than the remaining host Treg including a more stable expression of Foxp3*.* A therapeutic approach was initiated by infusing extra Foxp3^GFP^Treg together with the stem cells graft. These Treg expanded vigorously and were still present two months after the ASCT. However, by infusion of Treg the induction of stem cell-derived Treg was delayed and also the restoration of immune tolerance was impaired. These data indicate that restoration of the immune balance following ASCT depends on renewal of the natural Treg pool. Furthermore, infusion of Treg during ASCT seems to have a long-term negative effect on T cell reconstitution and re-establishment of immune tolerance.

